# Olivine-norite rock detected by the lunar rover Yutu-2 likely crystallized from the SPA-impact melt pool

**DOI:** 10.1093/nsr/nwz183

**Published:** 2019-11-14

**Authors:** Honglei Lin, Zhiping He, Wei Yang, Yangting Lin, Rui Xu, Chi Zhang, Meng-Hua Zhu, Rui Chang, Jinhai Zhang, Chunlai Li, Hongyu Lin, Yang Liu, Sheng Gou, Yong Wei, Sen Hu, Changbin Xue, Jianfeng Yang, Jie Zhong, Xiaohui Fu, Weixing Wan, Yongliao Zou

**Affiliations:** 1 Key Laboratory of Earth and Planetary Physics, Institute of Geology and Geophysics, Chinese Academy of Sciences, Beijing 100029, China; 2 Key Laboratory of Space Active Opto-Electronics Technology, Shanghai Institute of Technical Physics, Chinese Academy of Sciences, Shanghai 200083, China; 3 State Key Laboratory of Lunar and Planetary Sciences, Macau University of Science and Technology, Macau, China; 4 Beijing Institute of Space Mechanics and Electricity, Beijing 100076, China; 5 State Key Laboratory of Space Weather, National Space Science Center, Chinese Academy of Sciences, Beijing 100190, China; 6 State Key Laboratory of Remote Sensing Science, Institute of Remote Sensing and Digital Earth, Chinese Academy of Sciences, Beijing 100101, China; 7 Key Laboratory of Electronics and Information Technology for Space System, National Space Science Center, Chinese Academy of Sciences, Beijing 100190, China; 8 Xi’an Institute of Optics and Precision Mechanics, Chinese Academy of Sciences, Xi’an 710119, China; 9 Institute of Optics and Electronics, Chinese Academy of Sciences, Chengdu 610209, China; 10 Shandong Provincial Key Laboratory of Optical Astronomy and Solar-Terrestrial Environment, Institute of Space Sciences, Shandong University, Weihai 264209, China

**Keywords:** impact basin, lunar interior composition, visible and near-infrared spectra, lunar rover Yutu-2, Chang’E-4 mission

## Abstract

Chang’E-4 landed in the South Pole-Aitken (SPA) basin, providing a unique chance to probe the composition of the lunar interior. Its landing site is located on ejecta strips in Von Kármán crater that possibly originate from the neighboring Finsen crater. A surface rock and the lunar regolith at 10 sites along the rover Yutu-2 track were measured by the onboard Visible and Near-Infrared Imaging Spectrometer in the first three lunar days of mission operations. *In situ* spectra of the regolith have peak band positions at 1 and 2 μm, similar to the spectral data of Finsen ejecta from the Moon Mineralogy Mapper, which confirms that the regolith's composition of the landing area is mostly similar to that of Finsen ejecta. The rock spectrum shows similar band peak positions, but stronger absorptions, suggesting relatively fresh exposure. The rock may consist of 38.1 ± 5.4% low-Ca pyroxene, 13.9 ± 5.1% olivine and 48.0 ± 3.1% plagioclase, referred to as olivine-norite. The plagioclase-abundant and olivine-poor modal composition of the rock is inconsistent with the origin of the mantle, but representative of the lunar lower crust. Alternatively, the rock crystallized from the impact-derived melt pool formed by the SPA-impact event via mixing the lunar crust and mantle materials. This scenario is consistent with fast-cooling thermal conditions of a shallow melt pool, indicated by the fine to medium-sized texture (<3 mm) of the rock and the SPA-impact melting model [*Icarus* 2012; **220**: 730–43].

## INTRODUCTION

According to the Lunar Magma Ocean (LMO) hypothesis [1–3], the Moon was, early in its history, covered by global silicate magma up to 800 km thick [4], which crystallized into the modern crust and mantle. Several model compositions of the lunar mantle and crust have been proposed, with the lowermost cumulates dominated by Mg-rich olivine, followed by orthopyroxene (OPX) and finally by Ca pyroxene and plagioclase [5,6]. Furthermore, the lunar-mantle composition may have been modified by the overturn process, with the heavy top mantle materials sinking down due to gravitational instability [7]. In order to constrain the Moon's formation history, it is critically important to determine the composition of the lunar deep interior. The South Pole-Aitken (SPA) basin is the largest impact basin on the Moon, 2200 km in mean diameter and 13 km in depth [8,9], theoretically opening a window into the lunar lower crust and likely into the upper mantle [10–12]. However, compositional information of the SPA basin has mainly been obtained from orbital remote sensing [13–15] and there have been no *in situ* measurements of the region before the first landing on the far side of the Moon by Chang’E-4.

**Figure 1.**
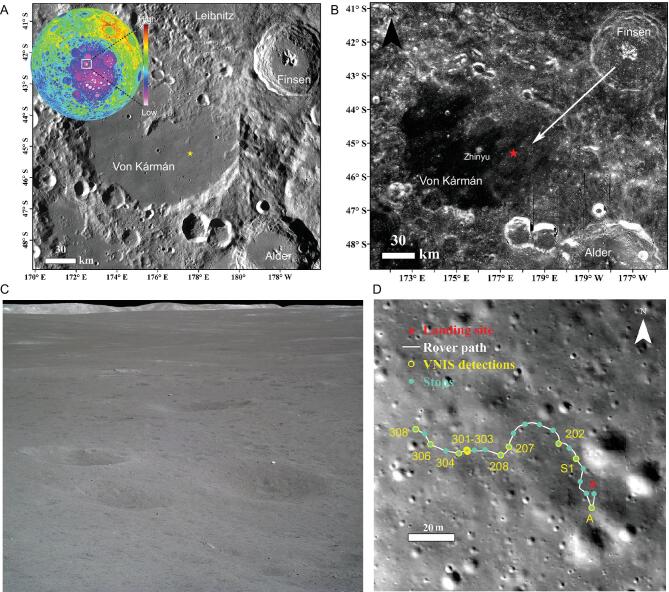
CE-4 landing site and the rover track. (A) The geologic setting of the CE-4 landing site (yellow star) seen in a Lunar Reconnaissance Orbiter Camera (LROC) Wide Angle Camera (WAC) image. The inset is SLDEM (SELENE and LRO DEM 2015) [16], stretched from -8000 to 10 000 m. (B) The ejecta strips radiating from Finsen crater. Zhinyu crater, the largest crater close to the landing site, is also labeled. (C) The typical landscape of the CE-4 landing site, showing very few rocks overlying the lunar regolith. (D) The Yutu-2 rover traverse during the first 3 lunar days of mission operations. The context image was taken by the descent camera on CE-4. The red star is the location of CE-4 landing site and the scale bar is 20 m.

On 3 January 2019, the Chinese lunar mission Chang’E-4 (CE-4), the backup of Chang’E-3 (CE-3), landed at 45.457°S, 177.588°E in Von Kármán crater (Fig. 1A), which is within the Mg-Pyroxene Annulus [13] of the SPA basin. Von Kármán crater has been filled with mare basalt and partially covered by ejecta from nearby impact craters. The landing site is located on ejecta strips radiating from Finsen crater, which lies ∼135 km to the northeast (Fig. 1B). The lunar surface at the landing site consists of very homogenous regolith overlain by a few scattered rocks (Fig. 1C). During the first three lunar days of mission operations, the Visible and Near-Infrared Imaging Spectrometer (VNIS) on board the rover Yutu-2 measured nine hyperspectral images of the lunar regolith and one hyperspectral image of a rock boulder along the 163-m rover track (Fig. 1D). These *in situ* measurements of the lunar regolith and overlying rock reveal the fine-scale composition of the SPA basin at unprecedented spatial resolution, shedding light on the composition of the lunar interior and the LMO crystallization.

## RESULTS

**Figure 2.**
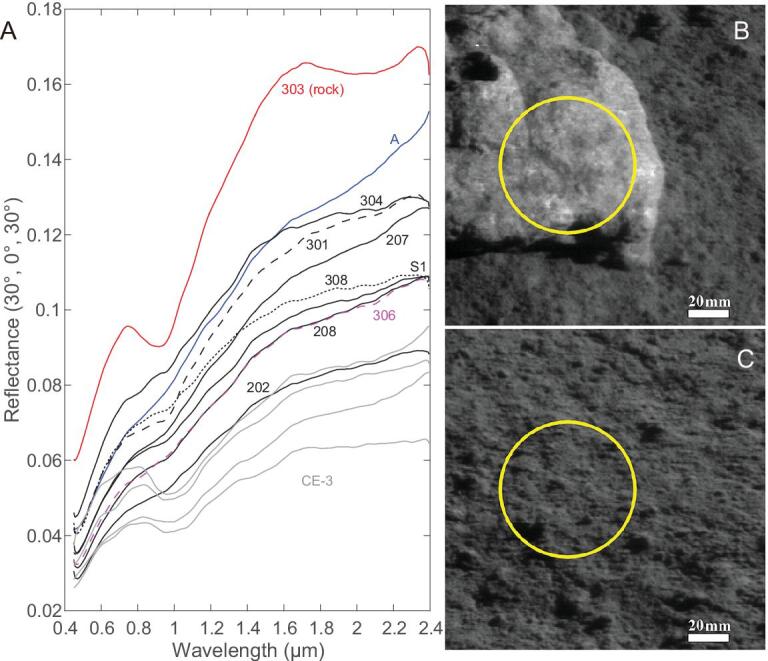
(A) VNIS spectra of the rock and lunar regolith measured by Chang’E-4. The analysis labels are the same as in Fig. 1D. Four other spectra (in gray color) of the basaltic lunar regolith measured by Chang’E-3 [17] are shown for comparison. (B) and (C) are the CMOS images of the rock (labeled as 303) and the lunar soil (labeled as 207) at 0.75 μm observed by VNIS imaging spectrometer, respectively. The yellow circle is the SWIR field and the scale bar is 20 mm.

The radiance spectra measured by VNIS on board Yutu-2 were converted to reflectance via division of the solar irradiance spectrum and then were photometrically corrected using an empirical photometric function (see ‘Materials and methods’). The spectra were smoothed twice with a boxcar average of 17 pixels followed by another boxcar average of 7 pixels. All 10 VNIS spectra, including 1 of the rock boulder and 9 of the lunar regolith, are shown in Fig. 2 alongside a close-up image of the rock and an image of the lunar regolith. The other images of the regolith are given in the Supplementary Materials (Supplementary Fig. 5). The surface around the CE-4 landing site is distinctly brighter than the basaltic soils at the Chang’E-3 landing site, which is exhibited by their different albedos (Fig. 2A).

**Figure 3.**
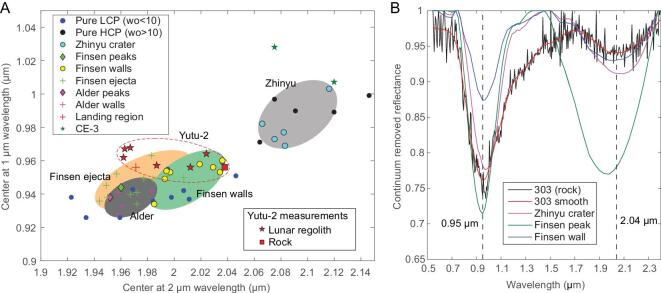
Spectral features of the lunar-surface materials observed by Yutu-2. (A) The peak center positions of the 1- and 2-μm bands, which are compared with those from spectra measured by CE-3 and the Moon Mineralogy Mapper (M^3^) of nearby craters. The pure LCP and HCP data are from [21]. The locations of all the M^3^ spectra are labeled in Supplementary Fig. 14. (B) Comparison of the rock spectrum with spectra from Zhinyu crater, Alder crater peak, Finsen crater peak and Finsen crater wall. The spectral calibration error of VNIS onboard CE-3 and CE-4 is <2 nm [22,23] and that of M^3^ is <1 nm [24].

The spectra of the lunar regolith and the rock show maximum absorptions at ∼1 and ∼2 μm (Fig. 2A and Supplementary Fig. 10). The peak positions of the ∼1- and ∼2-μm bands can be used to distinguish low-calcium pyroxene (LCP, centered at ∼0.95 and ∼1.9 μm) from high-calcium pyroxene (HCP, centered at ∼1.0 and ∼2.0 μm) [18] and to detect the possible presence of olivine (centered at ∼1.1 μm). The precise peak positions were determined by searching for the minimum reflectance [19] of the bands in continuum-removed [20] spectra (see ‘Materials and methods’). As shown in Fig. 3, the pyroxene composition of the lunar-surface materials measured by Yutu-2 is close to LCP, which is obviously different with the basalts excavated from Zhinyu crater.

The abundances of the constituents (i.e. agglutinates, total pyroxene, augite, olivine and plagioclase) of lunar regolith show strong correlation with the spectral parameters (empirical combination of albedos in the principal spectral bands) [25,26]. As such, we used a Lunar Soil Characterization Consortium (LSCC) dataset [27] to estimate the modal compositions of the regolith at the ChangE-4 landing site (see ‘Materials and methods’ and Supplementary Materials). The estimated results are summarized in Table 1, with an average modal composition of 55.9 ± 0.7% agglutinates (AGG), 13.5 ± 0.9% pyroxene (PYX) (4.9 ± 0.5% HCP, 8.6 ± 0.4% LCP), 13.6 ± 0.7% plagioclase (PLG) and 1.4 ± 0.3% olivine (OL). The maturity parameter I_s_/FeO can be derived from VNIS reflectance (Ref) spectra using equation S5 (0.0341 × Ref _500 nm_ – 0.2421 × Ref _750 nm_ – 0.0201 × Ref _900 nm_ + 0.2074 × Ref _1000 nm_ + 1.9430) [25,28] (see Supplementary Materials). The results are listed in Table 1.

**Table 1.** The estimated modal compositions and I_s_/FeO values of the lunar regolith measured by the Yutu-2 rover^a^.

Site	AGG	PYX	HCP	PLG	OL	I_s_/FeO	*D* (m)
Uncertainty	±7.7%	±3.1%	±1.3%	±4.4%	±0.8%	±18.8	–
A	57.1	11.5	3.8	15.0	1.6	97.0	11.5
S1	55.6	14.2	5.2	13.0	1.5	78.7	20.1
202	55.6	14.4	5.3	13.6	1.7	85.1	29.4
207	56.7	12.9	4.4	14.1	1.7	89.2	51.3
208	56.3	13.9	5.2	12.9	1.7	86.0	54.5
301	55.2	13.6	4.8	14.1	1.3	76.6	71.3
304	55.4	13.1	4.9	13.6	0.9	76.3	68.9
306	56.1	13.6	4.9	13.4	1.4	86.8	85.8
308	55.2	14.2	5.5	12.9	1.1	74.0	98.1

^a^AGG, agglutinates; PYX, pyroxene; HCP, augite; PLG, plagioclase; OL, olivine. *D* is the straight-line distance from the analysis position to the lander.

The rock analysed by Yutu-2 is >20 cm in size, sitting on the regolith surface. No grains can be unambiguously recognized on the surface, suggesting a fine- or medium-grain-size texture (<3 mm) based on the 0.6-mm/pixel spatial resolution of the image (Fig. 2B). In addition, the rock shows no lithic clasts of lunar-impact breccia (Supplementary Fig. 15), which usually vary in brightness and are embedded in a dark fine-grained matrix. The rock shows deep absorptions at ∼1- and ∼2-μm bands due to the low degree of space weathering. Hapke radiative transfer modeling [29], which has been validated and applied to lunar samples and meteorites [30,31] (see Supplementary Materials), was used to estimate the modal composition of the rock and our modeling results suggest 38.1 ± 5.4% LCP, 13.9 ± 5.1% olivine and 48.0 ± 3.1% plagioclase in this rock sample. As such, it is referred to as olivine-norite.

## DISCUSSION AND CONCLUSION

The spectral features at site A are distinct from those at other sites (Fig. 2A), resulting in different estimated modal mineral compositions and maturity (Table 1). Site A shows the highest agglutinates and I_s_/FeO value, indicating greater maturity than the other sites. Comparison to images acquired by the Lunar Reconnaissance Orbiter (LRO) Narrow Angle Camera (NAC) shows that the surface disturbed by CE-4 descent engines is about 1400 m^2^ (Supplementary Fig. 3), which is in agreement with the relationship between lander mass and blast-zone area constrained by previous missions [32]. Site A, about 11.5 m from the lander, should have been most significantly affected by the rocket exhaust. The higher maturity value at site A is therefore probably caused by deposition of the finest dust particles that were transported from the topmost surface below the lander blown by the rocket exhaust [33].

The regolith at the CE-4 landing site is dominated by plagioclase and pyroxenes with more LCP than HCP (1.8:1) (Table 1), consistently with the findings of a recent study [34]. The estimated modal composition of the lunar regolith is inconsistent with mare basalts (which would contain more pyroxene than plagioclase, with higher HCP than LCP) [35], but suggests its main source was norite [36]. Figure 3 also shows that band positions from *in situ* spectra of the lunar regolith at the landing site fall within the ranges of Finsen crater materials (the ejecta and crater walls), which are distinct from the underlying mare basalt excavated by Zhinyu crater (Supplementary Fig. 13) and the basaltic regolith on the nearside of the Moon that was analysed by Yutu-1 [17].

**Figure 4.**
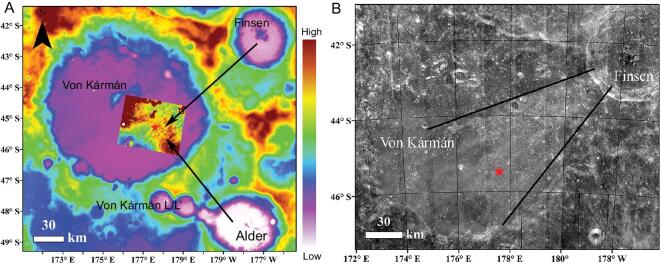
(A) SLDEM [16]. The image is stretched from -7581 to -158 m. The inset image is stretched from -6000 to -5900 m. (B) MI band ratio of 415 and 750 nm [39] (stretched from 0.53 to 0.55). The landing site is marked as a red star.

Our observations are also supported by topographic features. The landing site is located on NE–SW ejecta strips radiating from Finsen crater (Fig. 4), which superpose the SE–NW dome-like ridge directed towards Alder crater (Fig. 4A). Furthermore, the elevation difference between the landing site and the edge of the SE–NW dome-like ridge was estimated to be ∼70 m, suggesting a very thick deposit of ejecta from Finsen crater and probably also from Alder crater. Previous studies also reported that mare basalts were deeply buried by ejecta from nearby craters, with a minimum burial depth of 33 m [37,38]. Hence, the surface materials at the landing site are predominantly ejecta from neighboring craters, with little contribution from the underlying mare basalt.

The rock analysed by Yutu-2 is olivine-norite, not basalt. This is consistent with its setting on the surface of the regolith. Its modal composition is close to that of the regolith, but has higher modal abundances of olivine and LCP. It is possible that the rock was also delivered to its current location from Finsen crater. This is consistent with the similar peak positions of 1- and 2-μm bands (Fig. 3A). Alternatively, this rock may have been excavated from Alder crater. In this case, it would have been covered by ejecta from Finsen crater and then subsequently excavated from depth. However, this uncertainty makes no difference for constraining the composition of SPA basin, because both Finsen and Alder craters are located within the Mg-Pyroxene Annulus [13] at a similar distance (∼350 km) from the center of SPA basin. Hence, this rock is likely representative of the original bedrock in the Mg-Pyroxene Annulus of SPA basin.

**Figure 5.**
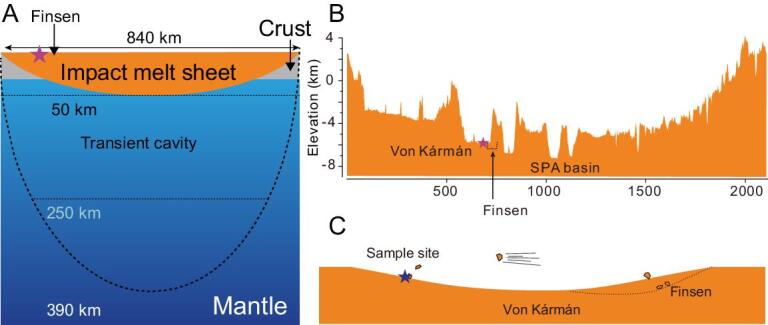
Schematic illustration for the origin of the rock and regolith analysed by Yutu-2. (A) The impact event of SPA formed a transient crater that was ∼840 km in diameter and ∼390 km in depth [10,43]. Hydrocode models suggest that the upper ∼250 km of the lunar interior was melted [43] and the melts gathered at the bottom of the SPA basin to form a ∼50-km-deep melt pond after impact [10,43]. (B) The topographic profile from A to A’ shown in Supplementary Fig. 2. (C) The schematic illustration of the sputtering process of the rock.

The SPA-impact event excavated the lunar deep interior and exposed lunar lower crust and/or mantle materials [10,12]. The bedrock of SPA basin could be the original plutonic rocks crystallized directly from LMO or may have formed from crystallization of the SPA-impact melt pool. The plagioclase-abundant and olivine-poor modal compositions of the materials measured by Yutu-2 rover are consistent with the origin of the lower crust rather than the mantle. The deep interior origin would also expect coarse-grained textures, typical of plutonic rocks (3 mm or larger) [40,41]. However, the observed fine- to medium-grain-sized texture of the rock suggests a fast-cooling thermal condition, which is consistent with crystallization from the SPA-impact melt pool [10,15,42]. Furthermore, the numerical simulations of impacting suggest that the SPA-scale event could generate a transient cavity with a diameter of 840 km and a melt pond with ∼50-km depth (Fig. 5A) [10,42,43]. The SPA-impact melt would have been a mixture of the lunar crust and upper-mantle materials. Both Finsen and Alder craters are located close to the margin of the melt pond (Fig. 5) [9,10]. Hence, the sources of the regolith and the rock boulder analysed by Yutu-2 are unlikely to be representative of the original lunar lower crust or the mantle as claimed by the previous study [44], but rather are the differentiated rocks from the impact melt pond. It is likely that the Finsen-impact event excavated shallow-layer materials crystallized from the SPA melt pond and delivered them to Von Kármán crater, where they were measured by the rover Yutu-2 (Fig. 5).

In summary, the *in situ* spectral measurements of the rock and regolith at the Chang’E-4 landing site result in similar estimates for mineral compositions and the rock is likely olivine-norite. These surface materials were delivered mainly from the neighboring Finsen crater, with possible additional contributions from Alder crater, but not from the underlying mare basalts. The fine- to medium-grain-size texture of the rock suggests fast crystallization, probably from the impact melt pond produced via melting the lunar lower crust and mantle materials by the SPA basin-forming event. This scenario is also consistent with the SPA-impact melting models [10]. These observations shed light on the composition of the SPA basin floor and the formation of the SPA basin.

## MATERIALS AND METHODS

### Data preprocessing

The VNIS on board the rover Yutu-2 consists of a Complementary Metal-Oxide Semiconductor (CMOS) imager (450–950 nm) with 256 × 256 pixels and a short-wavelength near-infrared (SWIR) detector (950–2395 nm) with single-pixel [22,45]. VNIS is installed on the front of the rover and measures the lunar surface from a height of ∼1 m above the lunar surface at a 45° emission angle. The SWIR field is centered at pixel 97.5, 127.5 of the CMOS field with a diameter of 107.6 pixels (Supplementary Fig. 1). The spectral-sampling interval of the CMOS and SWIR detectors is 5 nm, and the wavelength and FWHM (full width at half maximum) are shown in Supplementary Table 6.

The data were calibrated in flight and converted to reflectance using solar irradiance [46] and were then photometrically corrected [47,48] to common viewing geometry (incidence angle = 30°, emission angle = 0° and phase angle = 30°) based on illumination simulations conducted with lunar regolith simulants [49] (Supplementary Fig. 7).  The detailed description of this process is available in the Supplementary Materials.

The continuum is derived by fitting a convex hull over the top of a spectrum using straight-line segments (Supplementary Fig. 9) that connect local spectra maxima [20]. The band center position is defined as the minimum of a third-order polynomial fit to the channels of the band minimum [19]. All the Moon Mineralogy Mapper (M^3^) spectra and laboratory spectra used in this study were resampled to VNIS bands with a Gaussian model and the continuum-removed spectra were derived using the spectral range of 0.545–2.395 μm.

### Estimation of the mineral abundances from the regolith spectra

The statistical formulations linking spectral properties (empirical combination of spectral bands) with each mineral abundance were optimized with LSCC soils and have been successfully applied to Clementine UVVIS data [25,26]. We resampled the LSCC spectra to the spectral resolutions of Yutu-2 VNIS bands using a Gaussian model and parameterized the formulations (Supplementary Table 4) to estimate the mineralogy and maturity parameter of the regolith at the CE-4 landing site. The detailed descriptions are available in the Supplementary Materials.

### Estimation of the mineral abundance from the rock spectrum

The rock is much less affected by space weathering than the soil and has typical spectral characteristics. Thus, a more rigorous non-linear model, i.e. a Hapke radiative transfer model [29], was applied to retrieve the mineral abundances of the rock. The endmembers used in this study are high-calcium pyroxene (HCP), low-calcium pyroxene (LCP), olivine (OL) and plagioclase (PLG) (Supplementary Fig. 11 and Supplementary Table 5). More details about the methods are available in Supplementary Materials.

## Supplementary Material

nwz183_Supplemental_Files

## Data Availability

The data reported in this work will be archived at http://moon.bao.ac.cn/searchOrder_dataSearchData.search.
