# Supplementary material for: Olivine-norite rock detected by the lunar rover Yutu-2 likely crystallized from the SPA-impact melt pool
Source: Natl Sci Rev. 2019 Nov 14;7(5):913–20. doi: 10.1093/nsr/nwz183 (PMC8288882; doi:10.1093/nsr/nwz183)
Supplement: nwz183_Supplemental_Files [file nwz183_supplemental_files.zip › NSR_MS-2019-230-Supplementary Data.docx]

**Supplementary Materials**

**Olivine-norite rock detected by the lunar rover Yutu-2 likely crystallized from the SPA impact melt pool**

Honglei Lin^1†^, Zhiping He^2†^, Wei Yang^1^, Yangting Lin^1^*, Rui Xu^2^, Chi Zhang^1^, Meng-Hua Zhu^3^, Rui Chang^1^, Jinhai Zhang^1^, Chunlai Li^2^, Hongyu Lin^4^, Yang Liu^5^, Sheng Gou^6^, Yong Wei^1^, Sen Hu^1^, Changbin Xue^7^, Jianfeng Yang^8^, Jie Zhong^9^, Xiaohui Fu^10^, Weixing Wan^1^, Yongliao Zou^5^

^1^Key Laboratory of Earth and Planetary Physics, Institute of Geology and Geophysics, Chinese Academy of Sciences, Beijing 100029, China.

^2^Key Laboratory of Space Active Opto-Electronics Technology, Shanghai Institute of Technical Physics, Chinese Academy of Sciences, Shanghai 200083, China.

^3^State Key Laboratory of Lunar and Planetary Sciences, Macau University of Science and Technology, Macau, China.

^4^Beijing Institute of Space Mechanics and Electricity, Beijing 100076, China.

^5^State Key Laboratory of Space Weather, National Space Science Center, Chinese Academy of Sciences, Beijing 100190, China.

^6^State Key Laboratory of Remote Sensing Science, Institute of Remote Sensing and Digital Earth, Chinese Academy of Sciences, Beijing 100101, China.

^7^Key Laboratory of Electronics and Information Technology for Space System, National Space Science Center, Chinese Academy of Sciences, Beijing 100190, China.

^8^Xi'an Institute of Optics and Precision Mechanics, Chinese Academy of Sciences, Xi'an 710119, China.

^9^Institute of Optics and Electronics, Chinese Academy of Sciences, Chengdu 610209, China.

^10^Shandong Provincial Key Laboratory of Optical Astronomy and Solar-Terrestrial Environment, Institute of Space Sciences, Shandong University, Weihai 264209, China.

*Correspondence to: E-mail: [linyt@mail.iggcas.ac.cn](mailto:linyt@mail.iggcas.ac.cn).

†Equal contributions to this work.

**Supplementary Texts**

**Method for the reflectance determination.**

There are two methods to determine reflectance from the measurements of Visible and Near-Infrared Imaging Spectrometer (VNIS):

1) Using a calibrated near-Lambertian etalon, e.g. white panel

The Yutu-2 rover carried a white panel to allow onboard calibration. The absolute reflectance *Rs* (REFF) can be obtained by dividing the radiance *Is* of measured area to radiance *Ic* of white panel, which is a calibrated near-Lambertian etalon with known reflectance *Rc* (Table S1). The formula is:

$$R_{s}(i,e,\alpha)=\frac{I_{s}(i,e,\alpha)}{I_{c}(i,e,\alpha)}R_{c}(i,e,\alpha)$$

where, *i* is incidence angle, *e* is emission angle and *α* is phase angle.

2) Using solar irradiance

For airless planetary surface, the bidirectional reflectance are also commonly given as radiance factor (RADF) I/F (1):

$$I/F(\lambda_{i})=\frac{\pi I_{s}(\lambda_{i})}{\int J(\lambda)S(\lambda)d\lambda}$$

where J(λ) is the solar irradiance reaching the surface at 1 AU distance (2) and S(λ) is the spectral response of the VNIS sensor. I_s_(λ*_i_*) is the radiance measured by Yutu-2 at wavelength λ*_i_*. The relation between Rs and I/F is:

$$R_{s}=I/F/\cos(i)$$

The comparison between the reflectance obtained using white panel and solar irradiance at site A (the solar altitude is 39.123°) shows good agreement (Fig. S4), indicating the good radiometric calibration of the VNIS instrument.

The white panel in some points weren’t measured and such situation will be common in future detections of Yutu-2 rover based on engineering considerations. Moreover, the white panel at some sites was shadowed because of the low solar altitude. Thus, we use the reflectance determined using solar irradiance (Fig. S6**)**. All radiances *Is* of lunar surface used in this study have been calibrated in flight with the valid measurements of white panel (3). The CMOS reflectance spectra were scaled to their corresponding SWIR values using the overlapping region, because the SWIR detector can suppress background signal and have better quality.

**Method for the photometric correction.**

Photometric corrections are also needed due to the observations of same/different instruments are often measured under different geometrical conditions (incidence angle, emission angle and phase angle). Some models (4-6) were developed to correct orbital spectral data. However, these models can’t correct the observations with phase angle beyond 80 degree while most of the Chang’E-3/4 in-situ VNIS measurements have large phase angle (Table S2). Thus, we conducted an experiment that measured the bidirectional reflectance of synthetic lunar soil (7) (Table S3) using the backup VNIS instrument of CE-4 at phase angle from 20 to 110 degree, to obtain the phase curve of lunar soils in the laboratory. An empirical photometric function based on Lommel-Seeliger model was used (4):

$$RADF=\frac{\cos(i)}{\cos(i)+\cos(e)}f(\alpha)$$

A third order polynomial equation was derived to model the phase function *f(α)*. The exampled phase function of 900 nm, 1580 nm, 2000 nm and 2300 nm are shown in Fig. S7. Because of the lack of phase curve for CMOS bands, we corrected visible bands using the phase function at 900 nm. The corrected reflectance spectra are shown in Fig. S8.

**Estimation of the mineral abundances from the regolith spectra.**

The heavy space weathering attenuated the absorption features of lunar regolith and produced abundant glass-welded agglutinates by melting local materials driven by micrometeorite impacts, making it difficult to directly quantify the mineralogy from the regolith spectra. However, the abundances of the constituents (i.e. agglutinates, total pyroxene, augite, olivine and plagioclase) of lunar regolith have good mathematical links with the spectral parameters (empirical combination of albedos in the principal spectral bands) (8-10). Several statistical formulations describing links between mineral abundance and spectral bands were established using Lunar Soil Characterization Consortium (LSCC) soils ([http://www.planetary.brown.edu/relabdocs /LSCCsoil.html](http://www.planetary.brown.edu/relabdocs%20/LSCCsoil.html)) and successfully applied to Clementine data (8, 10). The VNIS instrument onboard Chang’E-4 covers the spectral range 450-2395 nm with 5 nm sampling interval, which is similar with the conditions of LSCC spectra measurements (300-2600 nm with 5 nm interval). Chang’E-4 acquired the spectra in an extremely high spatial resolutions (few centimeters) at a height of ~1 m above the lunar surface. So, the spectra between LSCC and Chang’E-4 are comparable. The LSCC dataset contains 9 mare and 10 highland samples, which provide the only ‘ground truth’ of lunar regolith with both VNIS spectra and known mineral abundance (11). Therefore, we resampled the LSCC spectra to the spectral resolutions of Yutu-2 VNIS bands using Gaussian model and parameterized the formulations linking mineral abundance with spectral albedos to estimate the mineralogy and maturity parameter of the regolith at the CE-4 landing site.

The formulation used for estimating the pyroxene abundance is the linear combination of albedo in the principal spectral bands (8):

$\log P=a_{1}A_{500}+a_{2}A_{750}+a{}_{3}A_{900}+a_{4}A_{1000}+a_{5}$ (S5)

where, *P* is the fractions of compositions and *A* is the spectral albedo (in %) at each wavelength. This equation is also used for estimating agglutinates abundance and Is/FeO value (8). The coefficients determined using LSCC dataset are summarized in Table S4.

The plagioclase has a uniquely broad absorption centered near ~1250nm, but weaken with the decrease of Fe content (12). Thus, the equation used to derive the fraction of plagioclase is modified as:

$\log P=a_{1}A_{500}+a_{2}A_{750}+a{}_{3}A_{900}+a_{4}A_{1000}+a_{5}A_{1100}+a_{6}A_{1250}+a_{7}$ (S6)

Using the LSCC dataset, the coefficients are determined as: a_1_ = 0.1540, a_2_ = -0.1644, a_3_ = -0.0534, a_4_ = 0.1517, a_5_ = -0.1580, a_6_ = 0.1142 and a_7_ = 1.0598. The correlation coefficient between the measured and estimated plagioclase of LSCC samples is 0.95 and the root mean square error is 4.4.

The olivine has characteristic absorptions centered at ~850nm, ~1050nm and ~1250nm (13, 14). Thus, the equation used to derive the fraction of olivine is modified as:

$\log P=a_{1}A_{500}+a_{2}A_{750}+a{}_{3}A_{850}+a_{4}A_{900}+a_{5}A_{1000}+a_{6}A_{1050}+a_{7}A_{1100}+a_{8}A_{1250}+a_{9}$(S7)

The coefficients in equation S7 are: a_1_ =0.2796, a_2_ = - 0.5925, a_3_ = 0.8411, a_4_ = - 0.7483, a_5_ = 0.7034, a_6_ = -1.2559, a_7_ = 0.8111, a_8_ = -0.0216 and a_9_ = 0.5008. The correlation coefficient between the measured and estimated olivine of LSCC samples is 0.74 and the root mean square error is 0.8.

The spectra of the soils in rover track were not analyzed because of the large relief and shadows, which have great influence on spectral characteristics.

**Estimation of the mineral abundance from the rock spectrum.**

The rock measured by Yutu-2 rover shows deep absorptions at ~1 and ~2 μm bands because of the low degree of space weathering. In this study, we use a Hapke radiative transfer model (15) because it is a more rigorous treatment of scattering on planetary surfaces. Hapke’s model is widely used in planetary remote sensing data, e.g. the Moon, Mercury and Mars. The Hapke’s model has been tested and applied to lunar samples (16) and meteorites (17). The mineral abundance errors are commonly within 5–10% (absolute error) (17).

- Surface Scattering Model

With the assumption that the particle size of mineral is much larger than the wavelength, the relations between reflectance and single-scattering albedo can be written as (15):

$r(i,e,\alpha)=\frac{w_{ave}}{4(\mu_{0}+\mu)}\{[1+B(\alpha)]P(\alpha)+H(\mu_{0},w_{ave})H(\mu,w_{ave})-1\}$ (S8)

where *r* is the reflectance, *μ_0_* and *μ* are the cosines of the angles of incidence and emission, respectively, *α* is phase angle.

B (*α*) is the backscattering function describing opposition effect:

 (S9)

where, $\varphi$ is filling factor, which is set to be 0.41 for the lunar regolith (18).

P (*α*) is the phase function, which can be expressed with Legendre polynomials (1):

$\begin{matrix} P(\alpha)=1+bP_{1}(\alpha)+cP_{2}(\alpha) \\ P_{1}(\alpha)=\cos(\alpha) \\ P_{2}(\alpha)=\frac{3}{2}\cos^{2}(\alpha)-\frac{1}{2} \end{matrix}$ (S10)

where, b and c are set as −0.4 and 0.25, respectively (19).

*w_ave_* is average single-scattering albedo, which can be computed as:

$w_{ave}=\sum_{i} \frac{M_{i}\omega_{i}}{\rho_{i}D_{i}}/\frac{M_{i}}{\rho_{i}D_{i}}$ (S11)

where, *M_i_*, *ρ_i_*, *D_i_* and *ω_i_* are mass fraction, density, particle size and single-scattering albedo of *i*th components, respectively (Table S5). The particle size of minerals on rock is assumed as 80 μm.

H is a multiple-scattering function (1):

$\begin{matrix} H(x,w_{ave})=\{1-(1-\gamma)x[r_{0}+(1-\frac{r_{0}}{2}-r_{0}x)\frac{\ln(1+x)}{x}]\}^{-1} \\ \gamma=\sqrt{1-w_{ave}},r_{0}=\frac{1-\gamma}{1+\gamma} \end{matrix}$ (S12)

- Single-Scattering Albedo of Mineral Endmember

The agglutinates are amorphous so that it’s difficult to determine the endmember spectrum. Thus, the endmembers used in this study are high-calcium pyroxene (HCP), low-calcium pyroxene (LCP), olivine (OL) and plagioclase (PLG) (Fig.S11), which are the dominant minerals of the Moon. We only focus on the relative fractions of these minerals. For laboratory endmembers, the single-scattering albedos should be derived using mineral optical constants. Single-scattering albedos of each endmember can be calculated using the Hapke model for given optical constants and grain size (1, 15):

$\omega=S_{e}+(1-S_{e})\frac{(1-S_{i})\theta}{1-S_{i}\theta}$ (S13)

$S_{e}=\frac{(n-1)^{2}+k^{2}}{(n+1)^{2}+k^{2}}+0.05$ (S14)

$S_{i}=1-\frac{4}{n(n+1)^{2}}$ (S15)

$\theta=e^{-\alpha<D>}$ (S16)

where, *n* and *k* are optical constants of the minerals. <*D*> is the average distance traveled by transmitted rays during one traverse of a particle:

$<D>=\frac{2}{3}[n^{2}-\frac{1}{n}(n^{2}-1)^{3/2}]D$ (S17)

where, D is particle size.

α=4π*nk*/*λ* is the absorption coefficient of the mineral. For considering the effects of space weathering to lunar surface, the Submicroscopic Metallic Fe (SMFe) was modeled to modify the absorption coefficient (16, 20):

$\alpha=\frac{4\pi nk}{\lambda}+\frac{36\pi zM_{Fe}\rho}{\lambda\rho_{Fe}}$ (S18)

$z=\frac{n^{3}n_{Fe}k_{Fe}}{(n_{Fe}^{2}-k_{Fe}^{2}+2n^{2})^{2}+4n_{Fe}^{2}k_{Fe}^{2}}$ (S19)

where, *n*, *k*, *ρ* are refraction indices and densities of host material, *n_Fe_*, *k_Fe_* and *ρ_Fe_* are refraction indices (21) and densities of SMFe.

Nonlinear least square method was used to solve Hapke radiative transfer model. The endmember spectra used in this study are listed in Table S5. The endmembers’ optical constant *k* can be first calculated using equation (S8)-(S17) for known reflectance and particle size (Table S5). Due to the thermal effects and low signal-noise ratio in the longer wavelengths, the spectra from 0.75-1.6 μm, which can represent the mixtures of the main minerals on the Moon, were used to derive the mineral abundance in this study. The model fits of in-situ spectrum of the rock boulder is shown in Fig. S12.


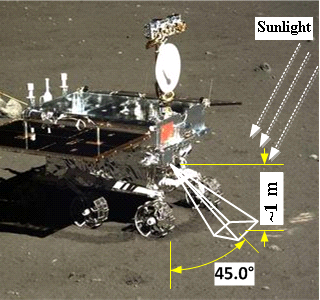

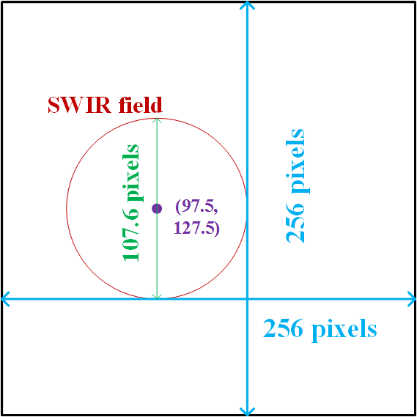


Fig. S1. Schematic diagram of the VNIS instrument working on the lunar surface and the field of view matching between CMOS (256×256 pixels) and SWIR detector (single pixel). The VNIS is installed on the front of the rover and measures the lunar surface from a height of ~1 m above the lunar surface at a 45° emission angle. The SWIR field is centered at pixel 97.5, 127.5 of the CMOS field with a diameter of 107.6 pixels.


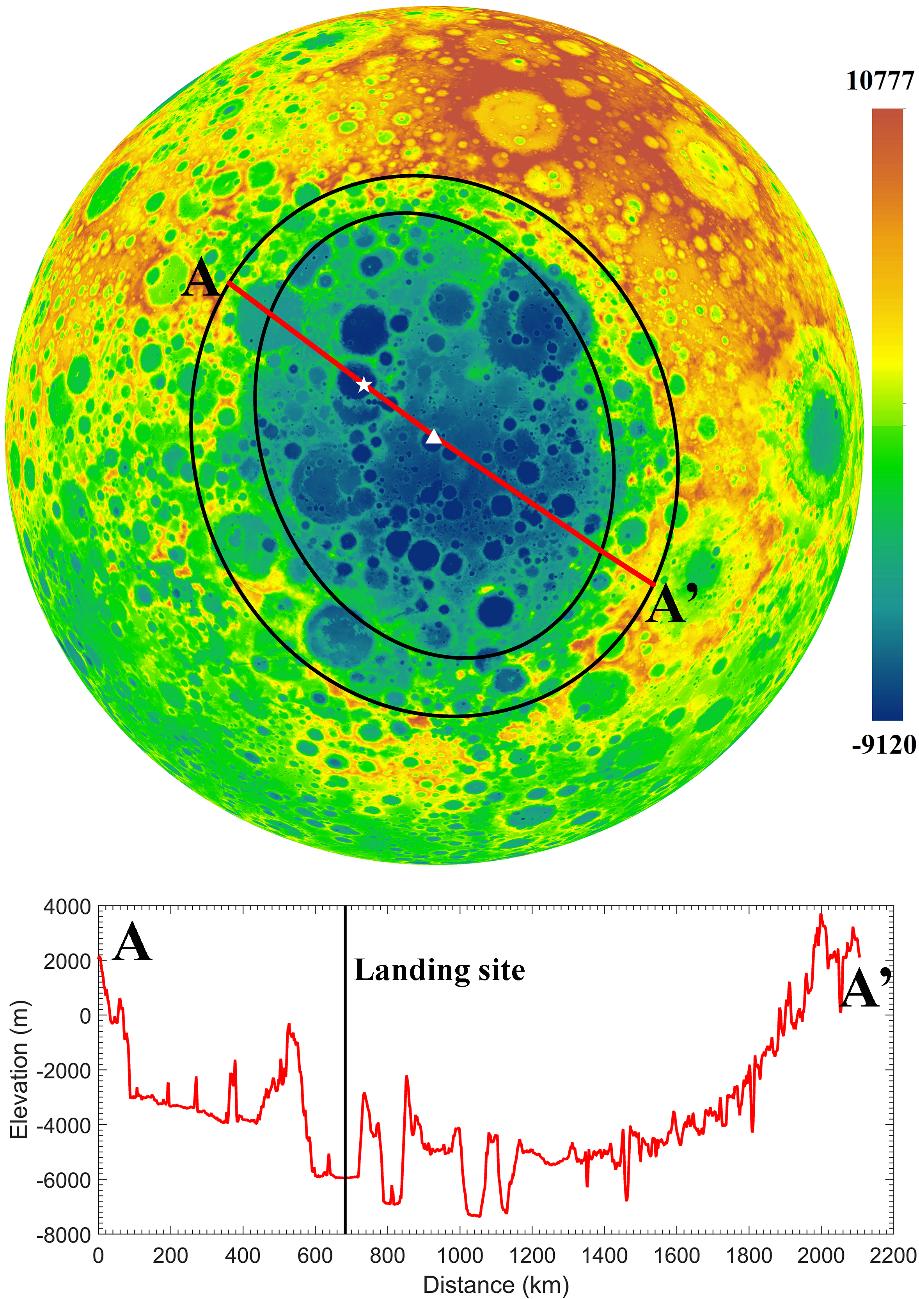


Fig. S2. The Orthographic projection of topography (unit: meter) of lunar farside as acquired by LOLA (22) and the elevation profile cross the SPA basin and CE-4 landing site. The LOLA elevation at landing site is about -5955 m and the distance from center of SPA basin (23) is ~370 km. The white star is the landing site of CE-4 mission and the white triangle is the center of SPA.


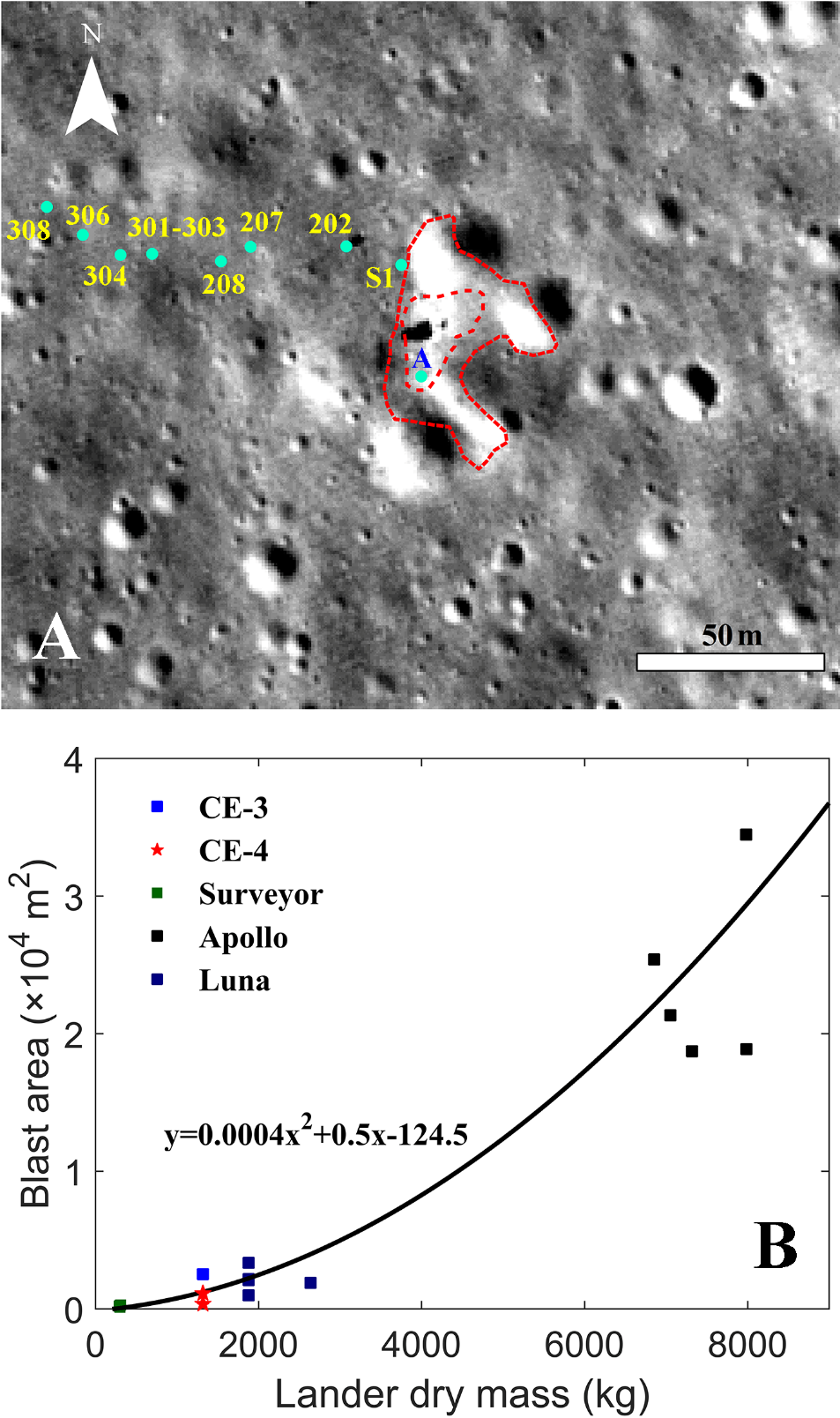


Fig. S3. (A) The blast zone of CE-4 landing site. The red regions are the possible blast areas with ~360 m^2^ and ~1140 m^2^, respectively. Some bright regions are possibly caused by the view geometry of LRO NAC. (B) Lander dry mass versus blast zone area (24).


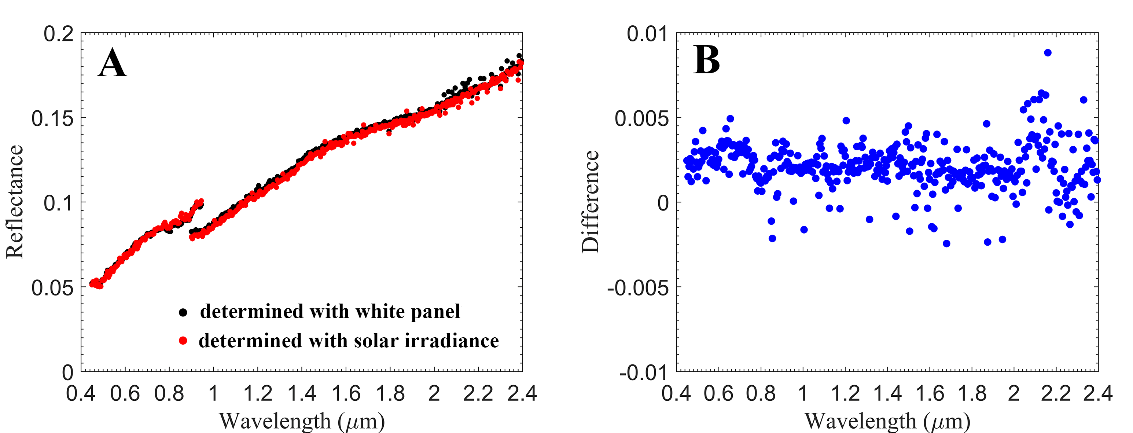


Fig. S4. (A) Reflectance values of lunar soil determined with white panel (black) and solar irradiance (red) of SWIR field at Site A. (B) The difference between reflectance obtained by two methods.


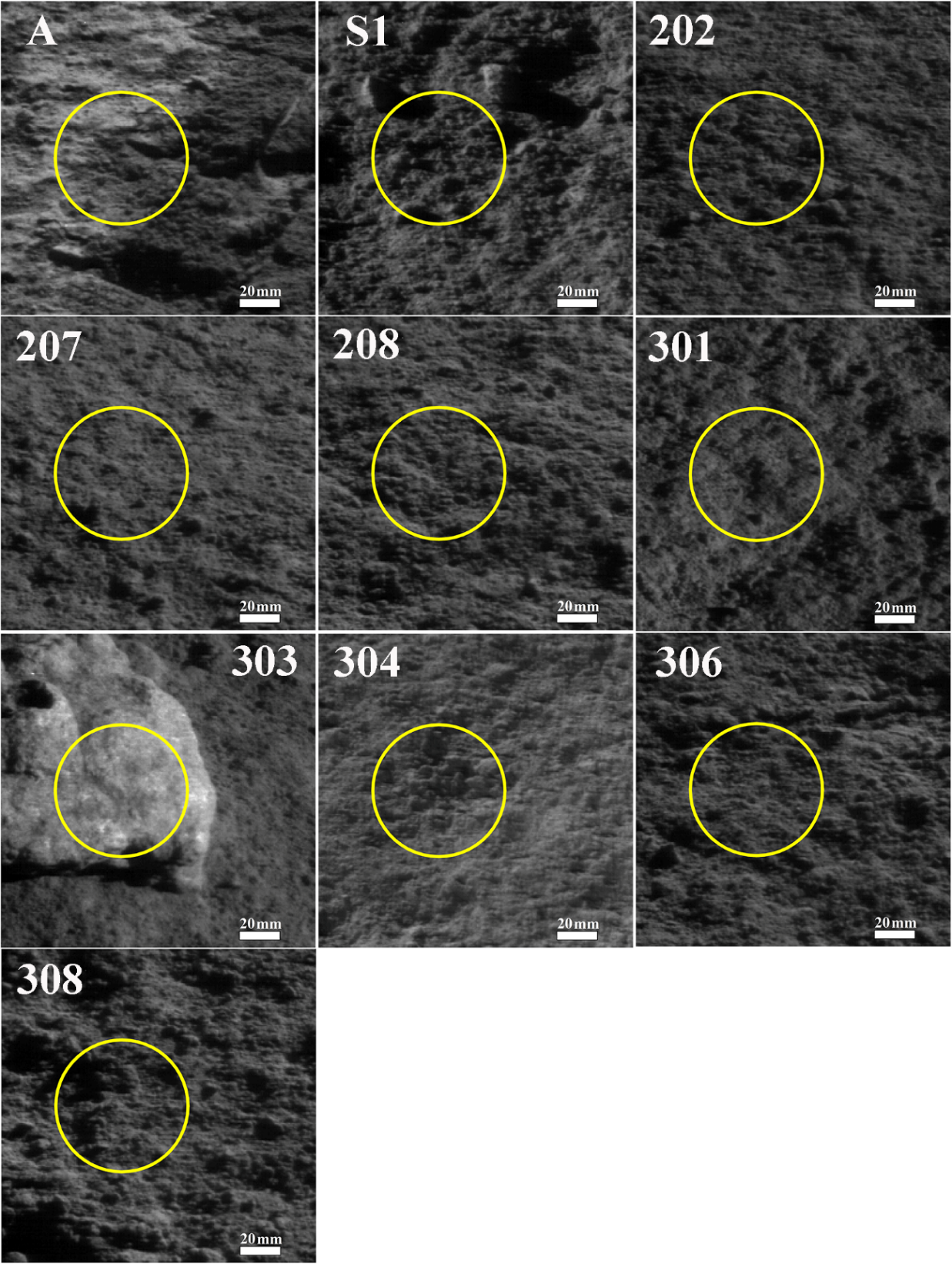


Fig. S5. The reflectance images of Yutu-2 measurements at 750 nm. All images are stretched from 0-0.25.


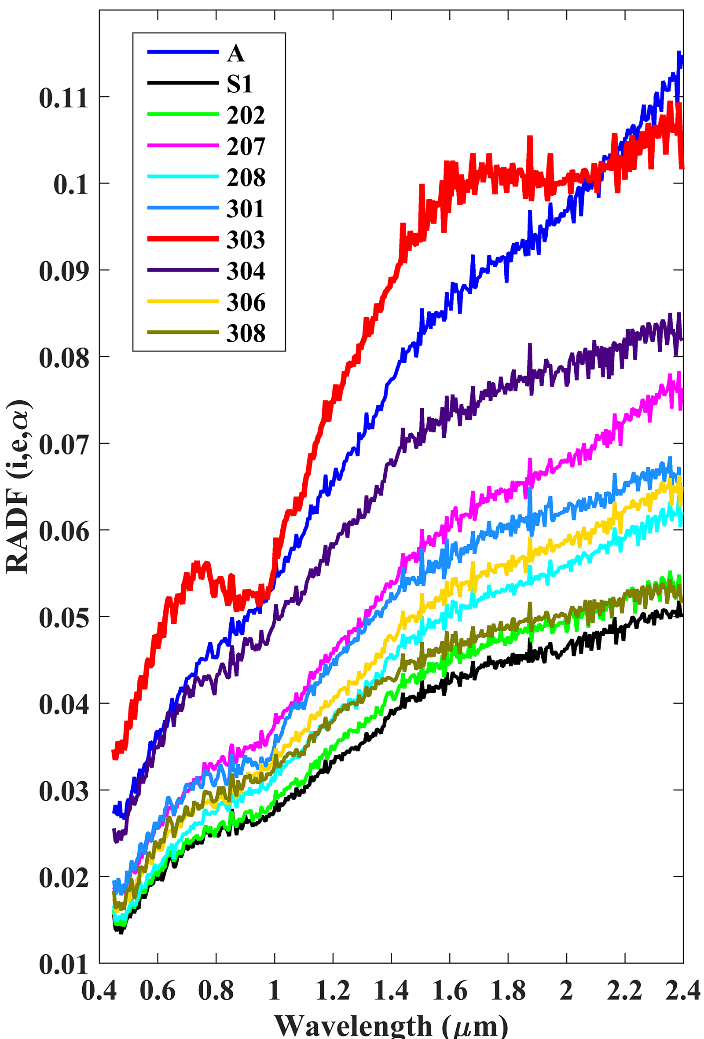


Fig. S6. The original reflectance spectra of lunar surface measured by CE-4 in the first three lunar days.


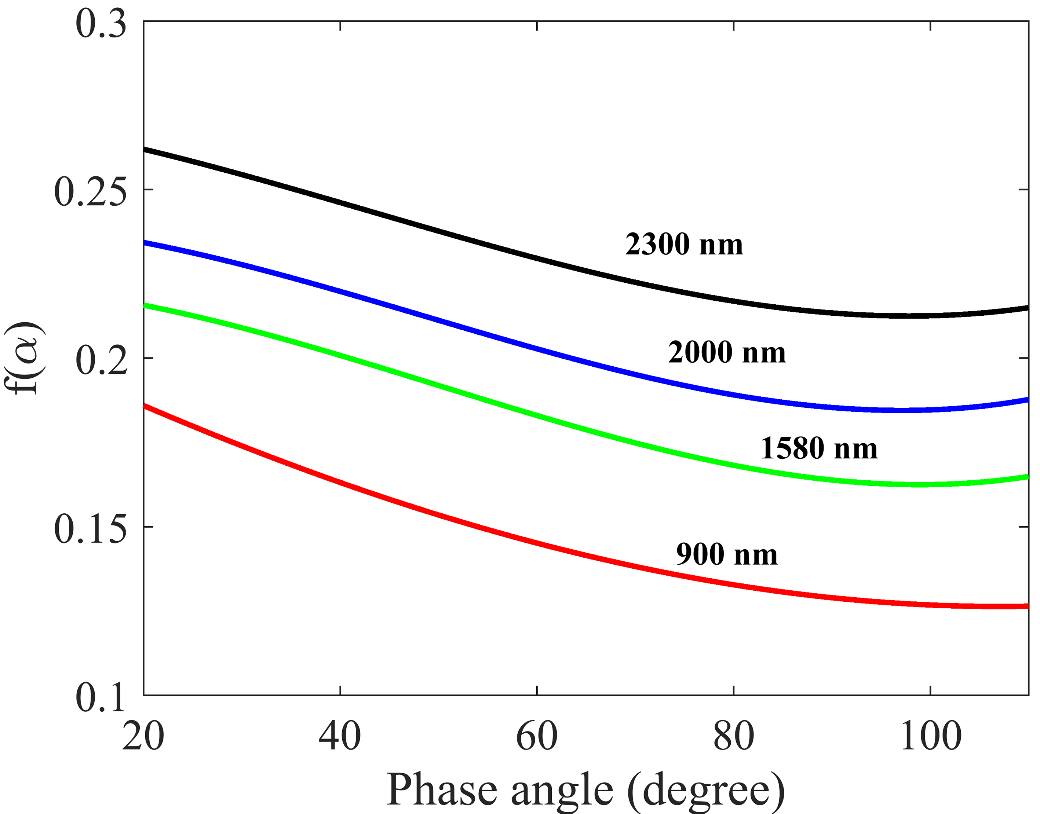


Fig. S7. Examples of phase function plots for wavelength of 900 nm, 1580 nm, 2000 nm and 2300 nm.


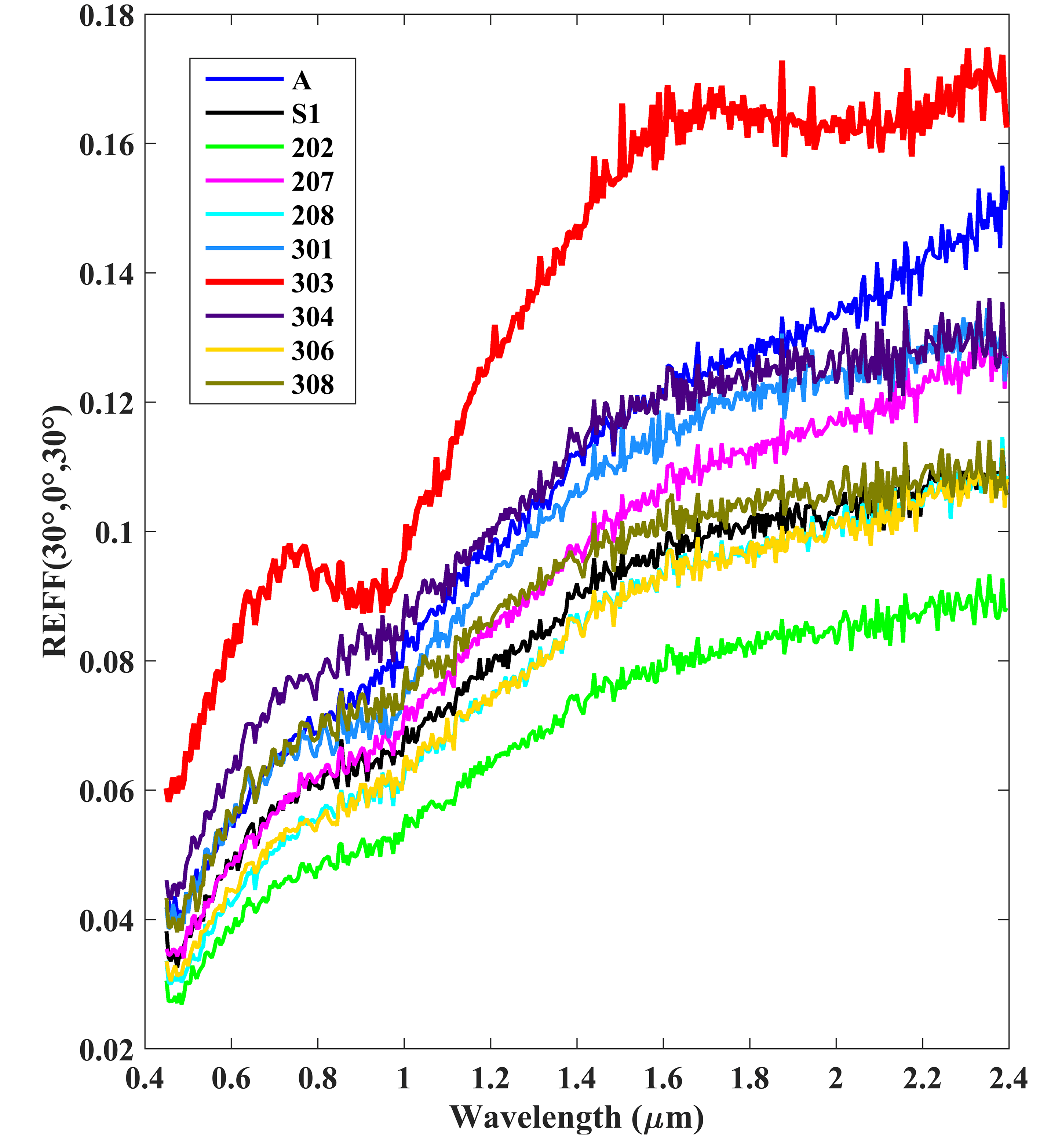


Fig. S8. The reflectance spectra, which are corrected to standard geometry (i=30°, e=0°, α=30°).


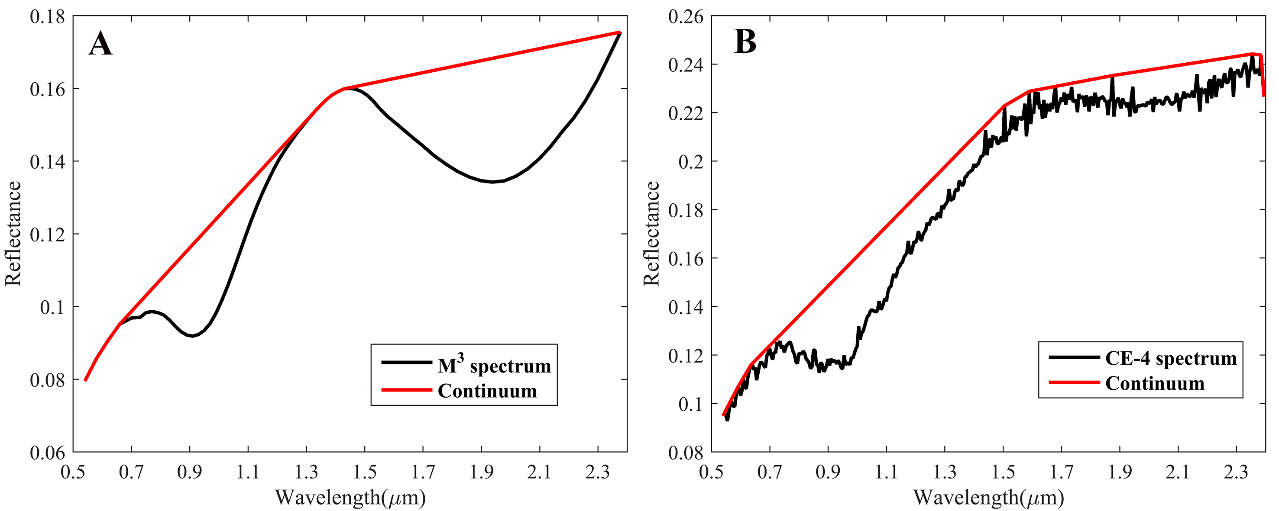


Fig. S9. The examples of continuum-removal.


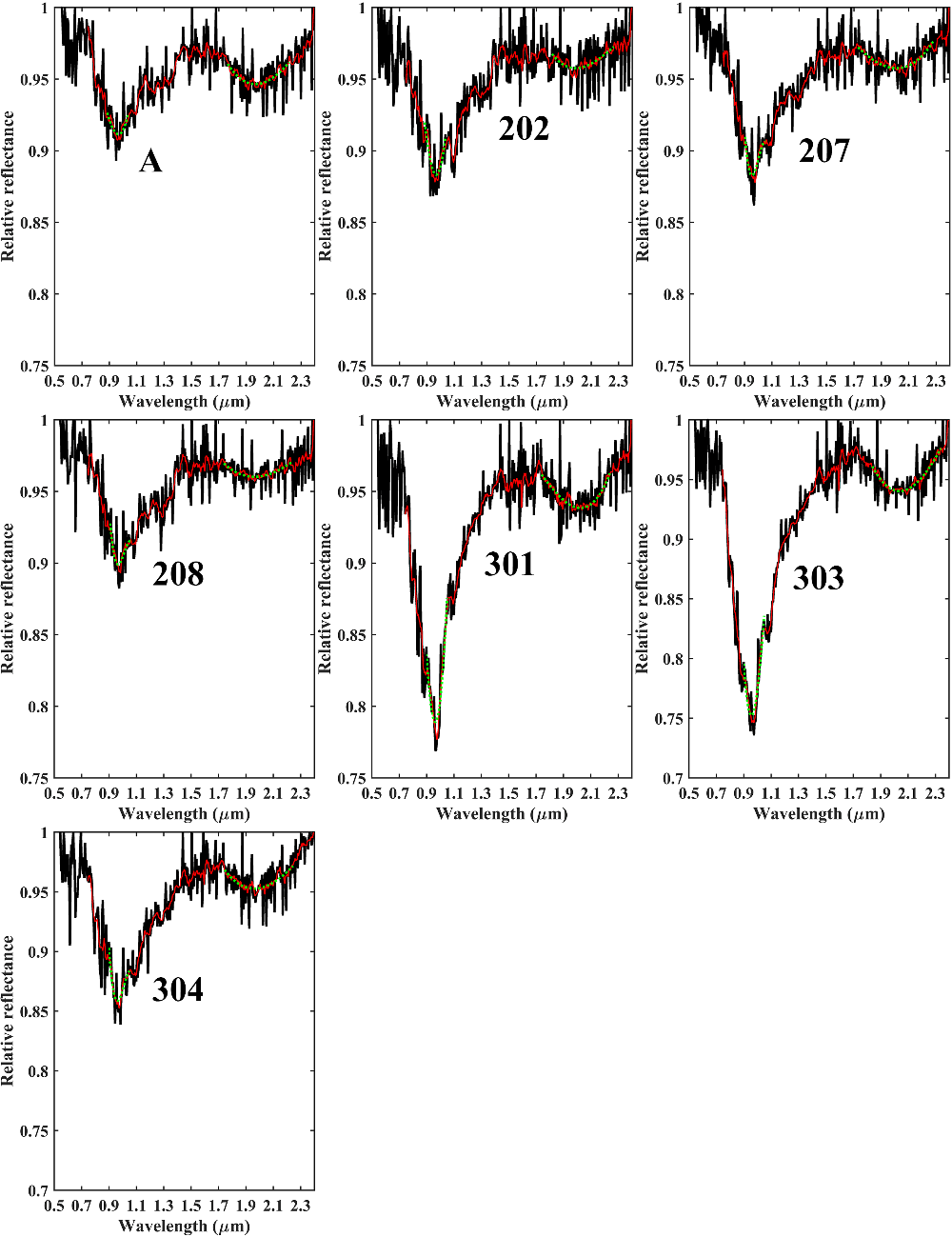


**Fig. S10.** The continuum-removed spectra of Yutu-2 measurements. The black lines are the continuum-removed spectra, red lines are the smoothed continuum-removed spectra and green dash lines are the third order polynomial fits of band centers. The band centers of sites S1, 306 and 308 weren’t calculated because of the weak absorption and too much noise at longer wavelengths.


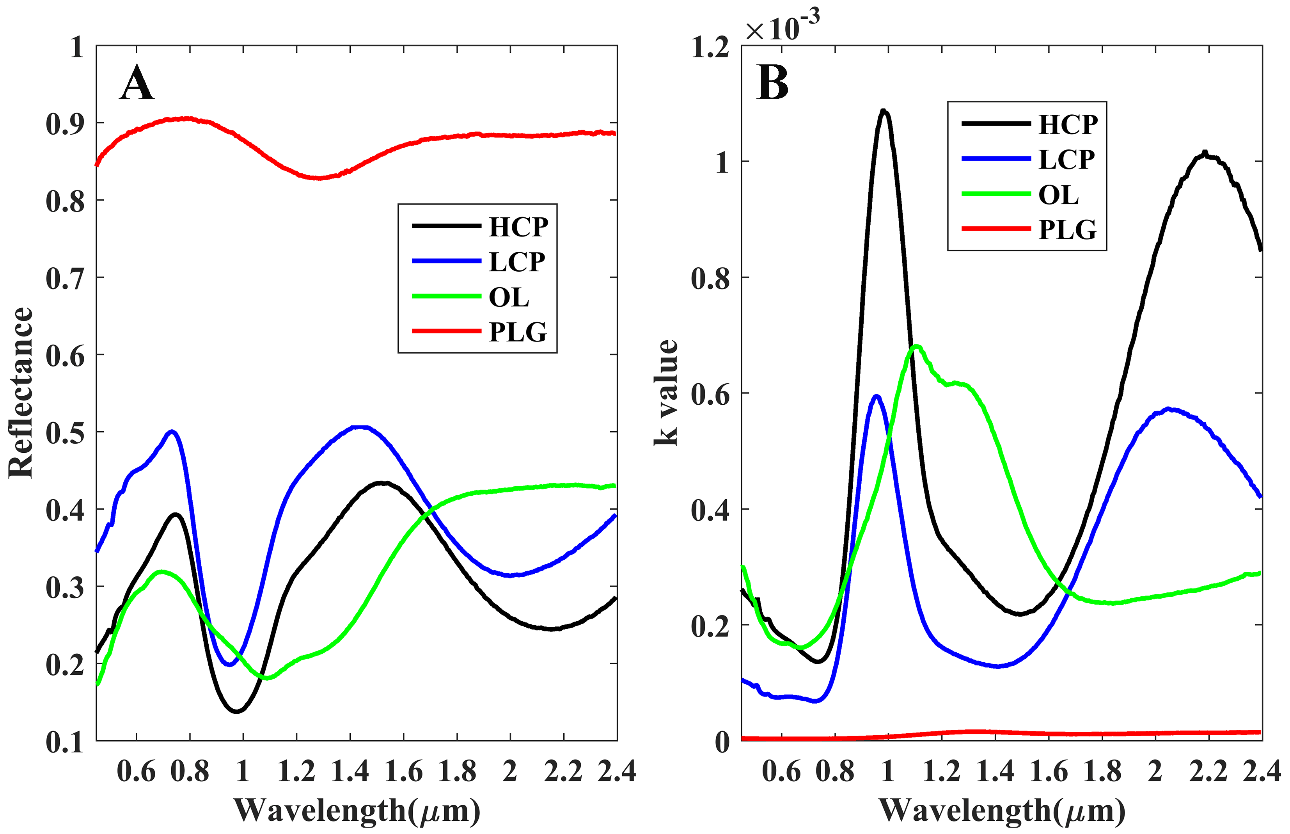


Fig. S11. Endmembers used for spectral modeling in this study. (A) Reflectance spectra of major endmembers. (B) The *k* value of all endmembers. The parameters used to derive *k* value are listed in Table S5.


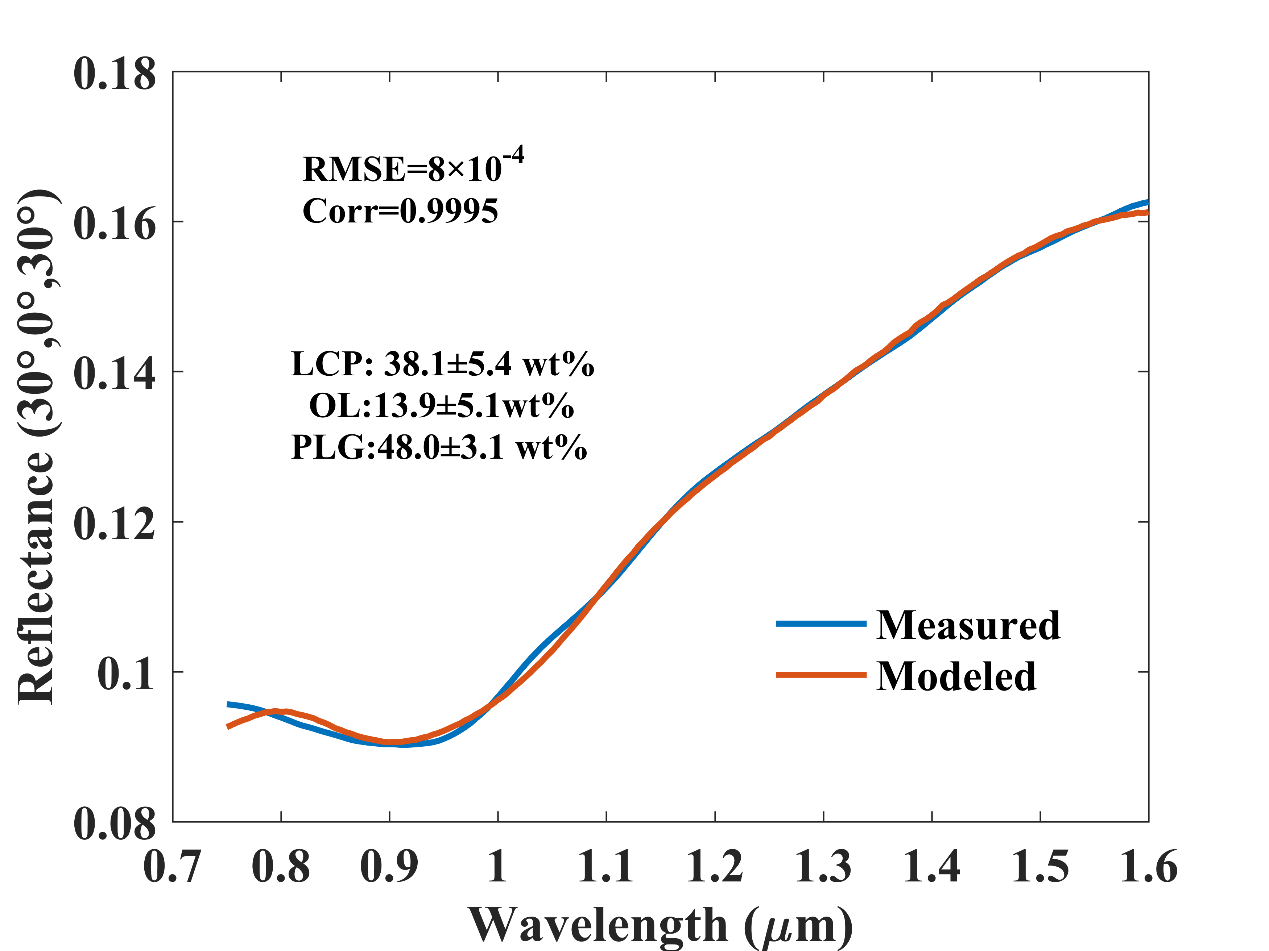


Fig. S12. Comparisons between modeled and measured reflectance spectra of the rock boulder, where RMSE is Root-Mean-Square Error and Corr stands for correlation coefficients.

**
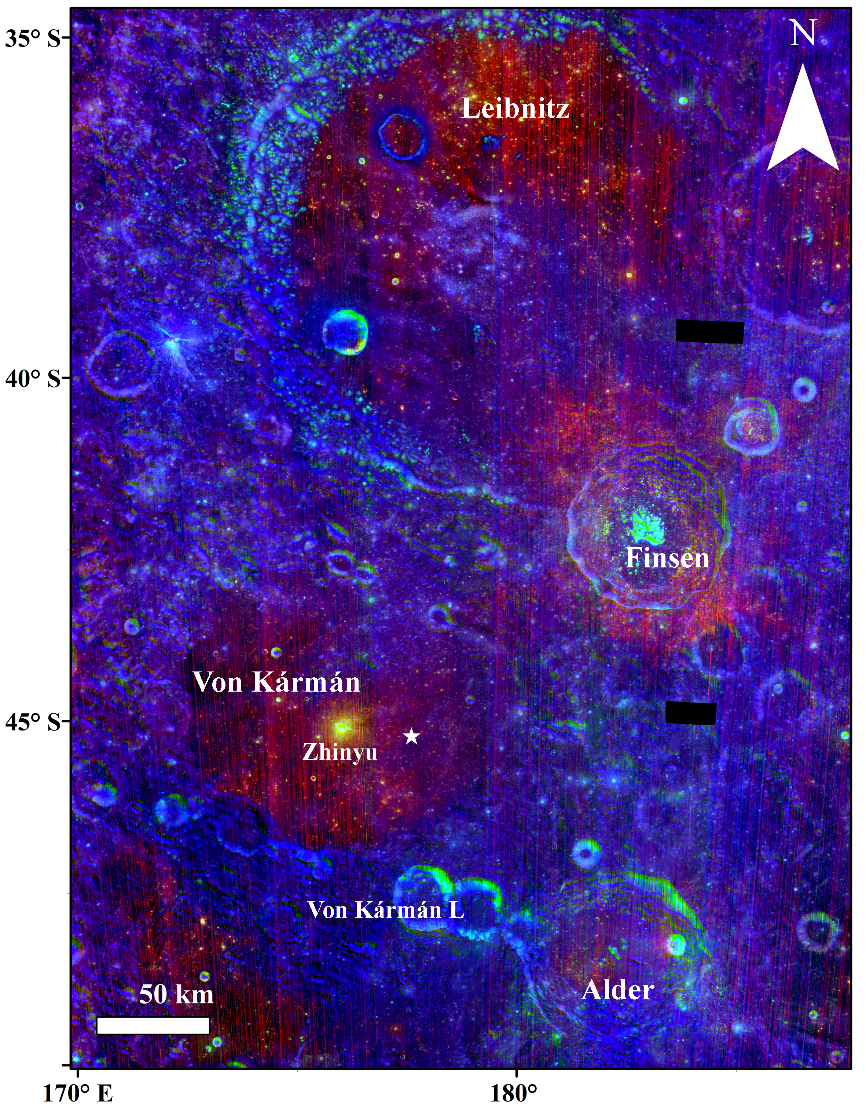
**

**Fig. S13.** Moon Mineralogy Mapper (M^3^) color composite with R=band center at 2 μm (stretch from 1.9 to 2.0); G=band depth at 2 μm (stretch from 0.04 to 0.17); B= reflectance at 1.58 μm (stretch from 0.08 to 0.155). Yellow regions suggest abundant HCP-bearing minerals and green-cyan regions suggest abundant LCP-bearing materials. The white star is landing site.


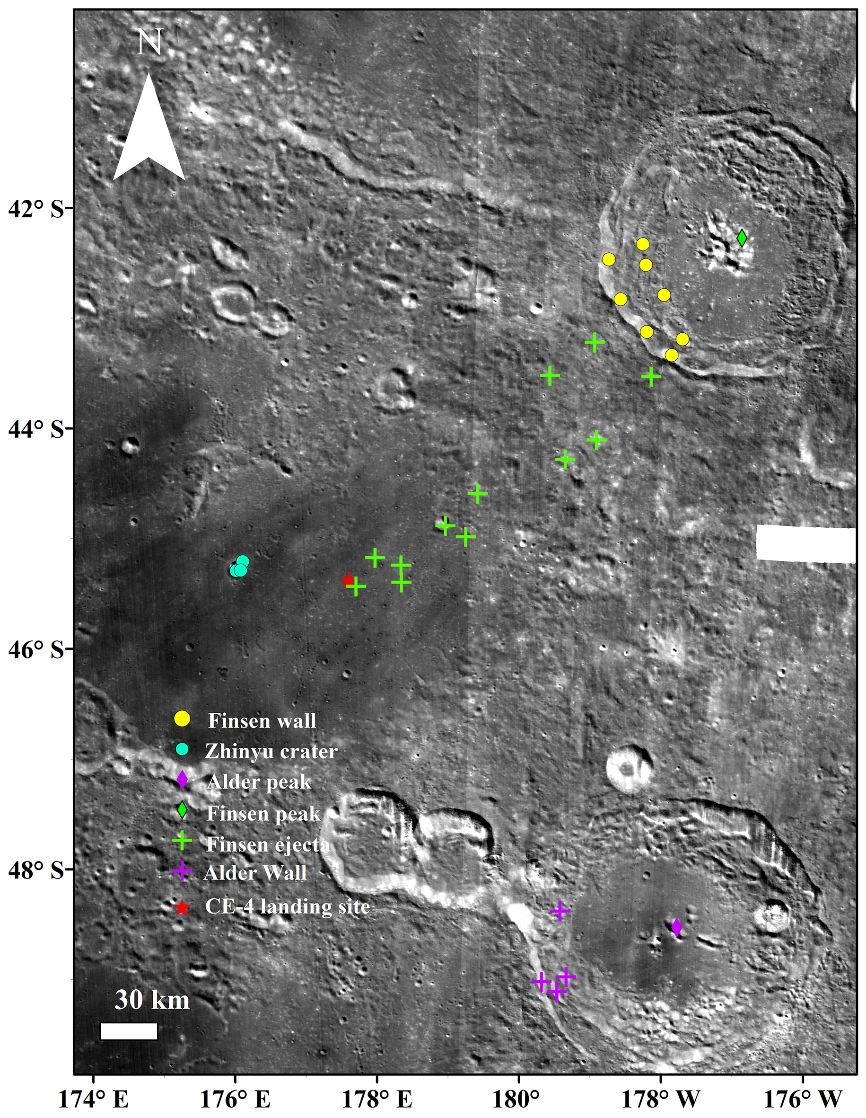


**Fig. S14.** The locations of the M^3^ spectra used in Fig. 3.

**
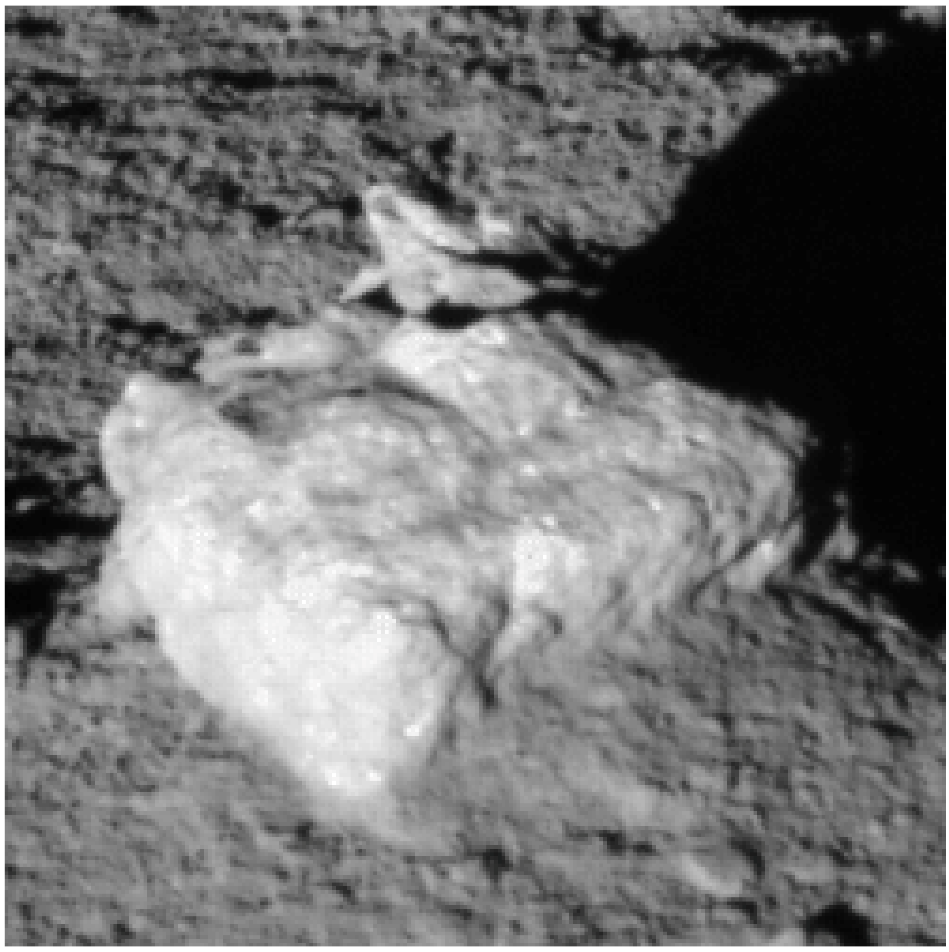
**

**Fig. S15.** Panoramic Camera image of the rock boulder measured by the VNIS instrument onboard Yutu-2 rover.

**Table S1.** The reflectance of white panel at different azimuth angles (-90º to 90º) and incidence angles (10º to 75º).

|  | -90° | –80° | –70° | –60° | –50° | –40° | –30° | –20° | –10° | 0° |
| --- | --- | --- | --- | --- | --- | --- | --- | --- | --- | --- |
| 10° | 0.997 | 1.000 | 0.999 | 1.006 | 1.006 | 1.006 | 1.009 | 1.024 | 1.002 | 1.016 |
| 15° | 0.995 | 1.001 | 1.001 | 1.010 | 1.012 | 1.014 | 1.019 | 1.037 | 1.017 | 1.028 |
| 20° | 0.991 | 0.998 | 1.001 | 1.013 | 1.016 | 1.021 | 1.028 | 1.051 | 1.032 | 1.040 |
| 25° | 0.987 | 0.995 | 0.999 | 1.013 | 1.020 | 1.028 | 1.039 | 1.066 | 1.049 | 1.056 |
| 30° | 0.983 | 0.994 | 0.999 | 1.014 | 1.025 | 1.036 | 1.051 | 1.083 | 1.071 | 1.077 |
| 35° | 0.979 | 0.992 | 1.000 | 1.016 | 1.029 | 1.044 | 1.064 | 1.104 | 1.100 | 1.108 |
| 40° | 0.974 | 0.987 | 0.997 | 1.017 | 1.034 | 1.051 | 1.073 | 1.122 | 1.127 | 1.138 |
| 45° | 0.971 | 0.984 | 0.998 | 1.018 | 1.038 | 1.058 | 1.084 | 1.141 | 1.154 | 1.174 |
| 50° | 0.965 | 0.979 | 0.997 | 1.020 | 1.042 | 1.067 | 1.096 | 1.157 | 1.176 | 1.199 |
| 55° | 0.960 | 0.976 | 0.993 | 1.022 | 1.046 | 1.073 | 1.106 | 1.173 | 1.195 | 1.218 |
| 60° | 0.952 | 0.971 | 0.990 | 1.020 | 1.047 | 1.077 | 1.114 | 1.185 | 1.207 | 1.232 |
| 65° | 0.950 | 0.968 | 0.990 | 1.022 | 1.052 | 1.089 | 1.129 | 1.204 | 1.231 | 1.249 |
| 70° | 0.938 | 0.959 | 0.980 | 1.017 | 1.053 | 1.089 | 1.131 | 1.218 | 1.244 | 1.265 |
| 75° | 0.925 | 0.943 | 0.969 | 1.008 | 1.050 | 1.087 | 1.131 | 1.224 | 1.252 | 1.273 |
|  | 10° | 20° | 30° | 40° | 50° | 60° | 70° | 80° | 90° |  |
| 10° | 0.973 | 0.978 | 0.980 | 0.983 | 0.985 | 0.985 | 0.986 | 0.984 | 0.985 |  |
| 15° | 0.986 | 0.991 | 0.991 | 0.991 | 0.990 | 0.989 | 0.989 | 0.984 | 0.984 |  |
| 20° | 1.001 | 1.004 | 1.001 | 0.999 | 0.995 | 0.993 | 0.990 | 0.981 | 0.980 |  |
| 25° | 1.018 | 1.018 | 1.013 | 1.007 | 1.001 | 0.995 | 0.989 | 0.980 | 0.975 |  |
| 30° | 1.040 | 1.035 | 1.026 | 1.017 | 1.008 | 0.999 | 0.990 | 0.978 | 0.972 |  |
| 35° | 1.066 | 1.057 | 1.041 | 1.029 | 1.016 | 1.003 | 0.992 | 0.978 | 0.970 |  |
| 40° | 1.093 | 1.077 | 1.055 | 1.038 | 1.021 | 1.006 | 0.992 | 0.976 | 0.965 |  |
| 45° | 1.126 | 1.101 | 1.072 | 1.049 | 1.029 | 1.010 | 0.994 | 0.976 | 0.963 |  |
| 50° | 1.157 | 1.123 | 1.089 | 1.060 | 1.036 | 1.014 | 0.996 | 0.975 | 0.959 |  |
| 55° | 1.188 | 1.146 | 1.106 | 1.072 | 1.043 | 1.018 | 0.996 | 0.974 | 0.955 |  |
| 60° | 1.215 | 1.167 | 1.121 | 1.082 | 1.050 | 1.020 | 0.995 | 0.971 | 0.950 |  |
| 65° | 1.247 | 1.192 | 1.143 | 1.099 | 1.060 | 1.026 | 0.999 | 0.970 | 0.947 |  |
| 70° | 1.271 | 1.216 | 1.159 | 1.106 | 1.066 | 1.027 | 0.997 | 0.966 | 0.940 |  |
| 75° | 1.293 | 1.235 | 1.173 | 1.115 | 1.069 | 1.026 | 0.991 | 0.957 | 0.927 |  |

Table S2. The viewing geometry of VNIS measurements in the first three lunar days.

| Number | Site | Time | *i* (°) | *e* (°) | *α* (°) |
| --- | --- | --- | --- | --- | --- |
| 1 | A | 2019-1-4 | 50.9 | 46.9 | 94.0 |
| 2 | S1 | 2019-1-11 | 72.1 | 42.9 | 89.5 |
| 3 | LE00202 | 2019-2-1 | 64.5 | 46.5 | 91.9 |
| 4 | LE00207 | 2019-2-9 | 64.6 | 46.3 | 90.8 |
| 5 | LE00208 | 2019-2-9 | 66.2 | 45.5 | 101.9 |
| 6 | LE00301 | 2019-3-1 | 70.9 | 43.3 | 66.9 |
| 7 | LE00303 | 2019-3-2 | 63.4 | 42.9 | 65.5 |
| 8 | LE00304 | 2019-3-2 | 62.1 | 47.5 | 105.4 |
| 9 | LE00306 | 2019-3-11 | 64.5 | 46.3 | 104.2 |
| 10 | LE00308 | 2019-3-12 | 71.4 | 45.3 | 102.5 |

Table S3. The information of synthetic lunar soil used in this study.

|  | Fractions |
| --- | --- |
| SiO_2_ | 49.507±0.090 % |
| Al_2_O_3_ | 15.803±0.114 % |
| FeO | 11.913±0.102 % |
| MgO | 8.715±0.088 % |
| CaO | 7.507±0.058 % |
| Na_2_O | 3.005±0.015 % |
| K_2_O | 1.750±0.005 % |
| MnO | 0.149±0.007 % |
| P_2_O_5_ | 0.499±0.011 % |
| TiO_2_ | 2.058±0.007 % |
| Ignition loss | 0.489±0.009 |
| Density | 2.820±0.008 g/cm^3^ |
| Particle >1 mm | 0.563±0.032 % |
| 0.1 mm<Particle <1 mm | 48.597±0.094 % |
| Particle < 0.1 mm | 50.810±0.066 % |

Table S4. Coefficients of Equation S5 determined using LSCC datasets. RMS (root mean square, σ) and correlation coefficient (k) are also listed.

|  | a_1_ | a_2_ | a_3_ | a_4_ | a_5_ | k | σ |
| --- | --- | --- | --- | --- | --- | --- | --- |
| Total PYX | -0.0501 | 0.1508 | -0.0494 | -0.1710 | 1.2766 | 0.89 | 3.1 |
| Augite | -0.0243 | 0.0715 | 0.3468 | -0.4051 | 0.8940 | 0.90 | 1.3 |
| AGG | -0.0271 | -0.0030 | -0.0210 | 0.0388 | 1.7371 | 0.77 | 7.7 |
| Is/FeO | 0.0341 | -0.2421 | -0.0201 | 0.2074 | 1.9430 | 0.92 | 18.8 |

Table S5. The endmembers used for spectral modeling in this work.

| Mineral | Spectra ID | *n* | Size range (μm) | Size used (μm) | density g/cm^3^ |
| --- | --- | --- | --- | --- | --- |
| HCP | LR-CMP-208 | 1.73 | 0-45 | 22 | 3.4 |
| LCP | LR-CMP-209 | 1.77 | 0-45 | 22 | 3.55 |
| OL | DD-MDD-041 | 1.83 | 0-45 | 22 | 3.32 |
| PLG | AG-TJM-011 | 1.56 | 0-45 | 22 | 2.68 |

**Table S6.** The wavelength and FWHM (full width at half maximum) of VNIS instrument onboard Yutu-2 rover.

Table S7. The IDs for the data used in this work.

| **Figure Number** | **Data ID** |
| --- | --- |
| Fig. 1D | CE4_GRAS_LCAM-1-2554_SCI_N_20190103022430_20190103022430_0001_A.2C |
| Fig.2A | CE4_GRAS_VNIS-VD_SCI_N_20190104004000_20190109213900_0001_A.2B |
|  | CE4_GRAS_VNIS-SD_SCI_N_20190104004000_20190109213900_0001_A.2B |
|  | CE4_GRAS_VNIS-VD_SCI_N_20190111150201_20190112102100_0003_A.2B |
|  | CE4_GRAS_VNIS-SD_SCI_N_20190111150201_20190112102100_0003_A.2B |
|  | CE4_GRAS_VNIS-VD_SCI_N_20190131080301_20190201054300_0005_A.2B |
|  | CE4_GRAS_VNIS-SD_SCI_N_20190131080301_20190201054300_0005_A.2B |
|  | CE4_GRAS_VNIS-VD_SCI_N_20190209053101_20190209130800_0008_A.2B |
|  | CE4_GRAS_VNIS-SD_SCI_N_20190209053101_20190209130800_0008_A.2B |
|  | CE4_GRAS_VNIS-VD_SCI_N_20190209130801_20190210033400_0009_A.2B |
|  | CE4_GRAS_VNIS-SD_SCI_N_20190209130801_20190210033400_0009_A.2B |
|  | CE4_GRAS_VNIS-VD_SCI_N_20190301065101_20190301155600_0012_A.2B |
|  | CE4_GRAS_VNIS-SD_SCI_N_20190301065101_20190301155600_0012_A.2B |
|  | CE4_GRAS_VNIS-VD_SCI_N_20190302060001_20190302131100_0015_A.2B |
|  | CE4_GRAS_VNIS-SD_SCI_N_20190302060001_20190302131100_0015_A.2B |
|  | CE4_GRAS_VNIS-VD_SCI_N_20190302131101_20190302160000_0016_A.2B |
|  | CE4_GRAS_VNIS-SD_SCI_N_20190302131101_20190302160000_0016_A.2B |
|  | CE4_GRAS_VNIS-VD_SCI_N_20190310092701_20190311064400_0020_A.2B |
|  | CE4_GRAS_VNIS-SD_SCI_N_20190310092701_20190311064400_0020_A.2B |
|  | CE4_GRAS_VNIS-VD_SCI_N_20190311064401_20190312052700_0021_A.2B |
|  | CE4_GRAS_VNIS-SD_SCI_N_20190311064401_20190312052700_0021_A.2B |

References

1. Hapke, B. *Theory of reflectance and emittance spectroscopy*: Cambridge university press; 2012.

2. Gueymard, CA. The sun's total and spectral irradiance for solar energy applications and solar radiation models. *Sol Energy*. 2004; **76**(4): 423-53.

3. Xu, R, Lv, G, Ma, Y-h*, et al.* Calibration of Visible and Near-infrared Imaging Spectrometer (VNIS) on lunar surface. In: *Multispectral, Hyperspectral, and Ultraspectral Remote Sensing Technology, Techniques and Applications V,* *2014*, p. 926315. International Society for Optics and Photonics.

4. Besse, S, Sunshine, J, Staid, M*, et al.* A visible and near-infrared photometric correction for Moon Mineralogy Mapper (M-3). *Icarus*. 2013; **222**(1): 229-42.

5. Wu, YZ, Besse, S, Li, JY*, et al.* Photometric correction and in-flight calibration of Chang' E-1 Interference Imaging Spectrometer (IIM) data. *Icarus*. 2013; **222**(1): 283-95.

6. Yokota, Y, Matsunaga, T, Ohtake, M*, et al.* Lunar photometric properties at wavelengths 0.5-1.6 mu m acquired by SELENE Spectral Profiler and their dependency on local albedo and latitudinal zones. *Icarus*. 2011; **215**(2): 639-60.

7. Zheng, YC, Wang, SJ, Ouyang, ZY*, et al.* CAS-1 lunar soil simulant. *Adv Space Res*. 2009; **43**(3): 448-54.

8. Pieters, C, Shkuratov, Y, Kaydash, V*, et al.* Lunar soil characterization consortium analyses: Pyroxene and maturity estimates derived from Clementine image data. *Icarus*. 2006; **184**(1): 83-101.

9. Shkuratov, YG, Kaydash, VG, Starukhina, LV*, et al.* Lunar surface agglutinates: Mapping composition anomalies. *Solar Syst Res+*. 2007; **41**(3): 177-85.

10. Shkuratov, YG, Kaydash, VG, Pieters, CM. Lunar clinopyroxene and plagioclase: Surface distribution and composition. *Solar Syst Res+*. 2005; **39**(4): 255-66.

11. Taylor, LA, Pieters, CM, Keller, LP*, et al.* Lunar Mare Soils: Space weathering and the major effects of surface-correlated nanophase Fe. *J Geophys Res-Planet*. 2001; **106**(E11): 27985-99.

12. Adams, JB, Goullaud, L. Plagioclase feldspars-Visible and near infrared diffuse reflectance spectra as applied to remote sensing. In: *Lunar and Planetary Science Conference Proceedings,* *1978*, p. 2901-9.

13. Sunshine, JM, Pieters, CM. Determining the composition of olivine from reflectance spectroscopy. *J Geophys Res-Planet*. 1998; **103**(E6): 13675-88.

14. Yamamoto, S, Nakamura, R, Matsunaga, T*, et al.* Possible mantle origin of olivine around lunar impact basins detected by SELENE. *Nature Geoscience*. 2010; **3**(8): 533-6.

15. Hapke, B. Bidirectional reflectance spectroscopy: 1. Theory. *Journal of Geophysical Research: Solid Earth*. 1981; **86**(B4): 3039-54.

16. Li, SA, Li, L. Radiative transfer modeling for quantifying lunar surface minerals, particle size, and submicroscopic metallic Fe. *J Geophys Res-Planet*. 2011; **116**.

17. Li, S, Milliken, RE. Estimating the modal mineralogy of eucrite and diogenite meteorites using visible-near infrared reflectance spectroscopy. *Meteorit Planet Sci*. 2015; **50**(11): 1821-50.

18. Bowell, E, Hapke, B, Domingue, D*, et al.* Application of photometric models to asteroids. In: *Asteroids II,* *1989*, p. 524-56.

19. Mustard, JF, Pieters, CM. Photometric Phase Functions of Common Geologic Minerals and Applications to Quantitative-Analysis of Mineral Mixture Reflectance Spectra. *J Geophys Res-Solid*. 1989; **94**(B10): 13619-34.

20. Hapke, B. Space weathering from Mercury to the asteroid belt. *J Geophys Res-Planet*. 2001; **106**(E5): 10039-73.

21. Johnson, P, Christy, R. Optical constants of transition metals: Ti, v, cr, mn, fe, co, ni, and pd. *Physical review B*. 1974; **9**(12): 5056.

22. Smith, DE, Zuber, MT, Neumann, GA*, et al.* Initial observations from the Lunar Orbiter Laser Altimeter (LOLA). *Geophys Res Lett*. 2010; **37**.

23. Garrick-Bethell, I, Zuber, MT. Elliptical structure of the lunar South Pole-Aitken basin. *Icarus*. 2009; **204**(2): 399-408.

24. Clegg-Watkins, R, Jolliff, B, Boyd, A*, et al.* Photometric characterization of the Chang’e-3 landing site using LROC NAC images. *Icarus*. 2016; **273**: 84-95.
